# Adult mental healthcare professionals’ experiences of family centred conversations with patients who are parents: a qualitative study

**DOI:** 10.3389/fpsyt.2024.1463823

**Published:** 2024-10-23

**Authors:** Louise J. Dalton, Simone de Cassan, Athif Ilyas, Abby Dunn, Elizabeth Rapa

**Affiliations:** ^1^ Department of Psychiatry, Warneford Hospital, University of Oxford, Oxford, United Kingdom; ^2^ Oxford Health National Health Service (NHS) Foundation Trust, Oxford, United Kingdom; ^3^ School of Psychology, Faculty of Health and Medical Sciences, University of Surrey, Guildford, Surrey, United Kingdom

**Keywords:** mental healthcare professionals, children, mental health, family centred conversations, effective communication

## Abstract

**Background:**

Many parents with a mental illness report a desire for both recognition of their parental role and support for themselves and their children. However, parents are often fearful of negative judgements from professionals about their ability to be a parent, which inhibits raising concerns about their children with clinical teams. Consequently, an essential first step to supporting families is for professionals to proactively identify which patients are parents, although evidence indicates this is not consistently part of services. Professionals could play a pivotal role in guiding parents about how to talk to their children about their mental illness; this in turn can facilitate family functioning and enable children to access evidence-based interventions. This approach is crucial for mitigating the well-documented intergenerational risk of poorer outcomes and mental illness in children of affected parents. This study aimed to explore adult mental healthcare professionals’ beliefs and experiences of talking with patients about: i) their role as parents ii) communication with their children about mental illness.

**Methods:**

Semi-structured qualitative interviews were conducted with 19 adult mental healthcare professionals with 4-30 years’ experience of working with adult patients under the care of NHS adult mental healthcare services in England. Audio recordings were transcribed verbatim and analysed using an inductive coding approach following the principles of thematic analysis.

**Result:**

All participants recognised their responsibility to identify patients’ children through routine safeguarding protocols, but supporting patients around telling their children the diagnosis was less common. Many participants expressed concerns that raising the topic of children with patients could conflict with other parts of their professional role or would not be welcomed. Obstacles to these conversations were identified, across individual, environmental and organisational factors. Potential facilitators included specific staff training and resources for clinical teams and families around how to tell children about mental illness.

**Conclusion:**

Adult mental healthcare professionals would benefit from enhanced training on the importance and methods of guiding parents to communicate with their children about mental illness. This should include routine identification of which patients are parents and recognition of the impact of an adult’s mental illness on the wider family system.

## Introduction

In the UK it has been estimated that 68% of women and 57% of men with mental health disorders are parents, the majority of which have depression and anxiety (1). A recent retrospective cohort analysis in the UK found that a quarter of children had a mother with a mental illness (2). This risk increased with children’s age, such that by the age of 16 years, there was a 53% chance of their mother being diagnosed with a mental disorder (2). A quarter of adults admitted to acute psychiatric inpatient units in the UK are reported to have dependent children (3). Data concerning the prevalence of paternal mental illness is woefully absent; however, one UK study reported that 21% of fathers had experienced depression by the time their children were 12 years old (4).

Children and adolescents of parents who have a mental illness are at increased risk of developing a mental illness themselves (5–7). In addition, being a child of a parent with a mental disorder is associated with other psychological and developmental risks including behavioural problems (8), attentional difficulties (9), poorer cognitive development (10), disorganised attachment (11), emotional dysregulation (12), self-harm and suicide attempts (13). While genetic mechanisms play a part, it is well established that the impact of mental illness on parenting and family relationships are a key pathway to these adverse outcomes (14, 15). However, it is important to note that not all children who have a parent with mental health problems develop psychological difficulties (16); protective factors include parental positive expressed emotion, good quality social relationships, self-efficacy and frequent exercise with evidence of better mental health outcomes for adolescents with more protective factors (17).

A key approach in supporting families affected by mental illness may be to focus on facilitating communication with children about their parent’s diagnosis. A challenge facing many families is the issue of ‘if’ and ‘how’ to tell children about parental illness. Parents with mental health difficulties often feel that their child will not understand their illness (18), which risks contributing to a belief that mental health disorders are a source of shame and must be kept hidden (18, 19). When children are not given an explanation of their parent’s illness, their understanding may be based on their observations, which can perpetuate worries and misconceptions (20). Children’s comprehension of illness evolves during childhood, so it is essential that communication is appropriately matched to their developmental understanding (21). Effective communication about a parent’s illness can help children understand their parent’s symptoms (22), bring a sense of relief and mitigate feelings of blame (23, 24). Communication also helps children develop a language to talk to others (24). This may help them access social and emotional support before difficulties escalate (24). Furthermore, when families can talk together about challenges and distressing experiences, it helps strengthen their family connectedness (25) and in turn enhances resilience (26).

Research consistently indicates that many patients fear their children will be removed from their care, leaving them reluctant to share concerns or request support from mental health services regarding their parenting (27–29). However, adult patients do want support in their role as a parent and recognise the importance of helping their children cope with having a parent with a mental illness (30). Understanding mental health and parental symptoms are identified by both parents and children as an important element of this support (30–33). Interventions have been developed to support patients talking to their children about their mental illness, with evidence indicating these can have positive effects on children’s outcomes including internalising symptoms (34), increased illness knowledge and decreased shame (35, 36), as well as improved family communication (25). The identification of patients with children can in turn enable these parents to access interventions to support their parenting and family relationships, which can have benefits for both parental and family outcomes (37, 38).

For the children of adults with a mental illness to benefit from these interventions, patients must first be identified as being a parent or caregiver. The UK Care Act 2014 outlines the expectation that healthcare professionals should routinely record details of every patient’s responsibility for dependent children. However, eight years on from the launch of this legislation, national evidence indicates that this expectation is not being met (39). A recent survey of over 1000 adult mental healthcare practitioners in England found that a quarter did not routinely identify patients as parents (39). Furthermore, less than one third of respondents routinely asked patients if their children had any emotional or behavioural difficulties (39). These findings are consistent with a previous audit of adult mental health services reporting only 62 out of 100 cases were asked if they had dependent children (40). This body of evidence reflects research documenting that the absence of policy and guidelines, inadequate resources, high workloads and a perception that children are not the responsibility of adult services hinder professionals identifying parents (41). It has been well documented that broader systemic changes are needed, including organisational commitments (42) and opportunities for professionals to reflect on developing and maintaining relationship-based practice with patients (43). However, at the point of care identifying patients as parents is an essential first step to initiating a discussion about whether their children have been told about a parent’s mental illness and what support may be needed.

The aim of this study was to explore mental healthcare professionals’ (MHPs) beliefs and experiences of talking with patients about their role as parents and parents’ communication with their children about mental illness.

The objectives were to investigate:

1. adult mental healthcare professionals’ perceptions of their role in identifying patients as parents.

2. adult mental healthcare professionals’ perceptions of their role in supporting parents to communicate with their children (family-centred conversations) about mental illness.

3. adult mental healthcare professionals’ perceptions of the barriers and facilitators in supporting parents to communicate with their children (family-centred conversations) about mental illness.

## Material and methods

This exploratory qualitative study used semi-structured in-depth interviews and is reported in accordance with the Consolidated Criteria for Reporting Research checklist for qualitative research (44).

### Ethical approval

Ethical Approval was granted by the University of Oxford Medical Sciences Interdivisional Research Ethics Committee (Reference: R69965/RE001). All participants provided informed consent prior to taking part. All methods were performed in accordance with the relevant guidelines and regulations. No participant withdrew from the study. All potentially identifying information about participants was removed from transcripts to protect participants’ confidentiality.

### Eligibility and recruitment

Participants were eligible if they were over the age of 18, registered mental healthcare professionals employed by the National Health Service (NHS) and had experience of working with patients over the age of 18 who had mental health difficulties and were also parents. Using convenience and volunteer sampling techniques, 20 MHPs were recruited between August 2021 and December 2021. Study adverts were developed by the research team and distributed via email to their existing networks inviting participants to take part in a one-off interview. Participants were provided with a Participant Information Sheet which included the rationale for the study and the research team’s interest in this topic. Interested participants directly contacted the research team for further information and agreed a date and time for the interview. One professional contacted the study team but did not take part in the research due to time commitments.

### Data collection

Nineteen participants gave informed consent for participation in the study and inclusion of written quotations in the publication of the project findings. Interviews were conducted by two researchers, SdC and ER, with the majority being conducted by SdC. The interviewers had no prior relationships with the participants they interviewed. A semi-structured interview guide was developed, informed by the study’s aims, objectives and the expertise of the research team (**Table 1**). The guide was iteratively reviewed throughout the study. All interviews were conducted via Microsoft Teams and audio recorded; no field notes were taken. Interviews lasted between 16.5 and 55.1 minutes (mean = 47.1 minutes) and there were no repeat interviews. No non-participants were present during the interviews. Data collection was terminated once no further categories were identified.

**Table 1:** Semi-structured topic guide.

1. Exploration of MHPs’ usual practice about identifying patients who are parents
2. MHPs’ experience of talking to patients about what their children know about their diagnosis
3. Perceptions of what underpins patients’ attitudes and behaviour about talking to their children
4. MHPs’ perception of facilitators and obstacles to family centred conversations

### Data analysis

Audio-recordings were transcribed verbatim by SdC; transcripts were not returned to participants for correction or comments. Data were analysed using an inductive coding approach following the principles of reflexive thematic analysis using NVivo 12 (45, 46). Initially SdC, AI, ER and LJD read and familiarised themselves with the transcripts, noting and recording initial themes derived from the data, which were refined by discussions with the research team. Themes were identified based on the list of codes and organised into themes with the help of mind-mapping activities and discussion with the research team. Participants were not invited to provide feedback on the findings.

### Reflexivity, research group and context

SdC identifies as a white female and is a Psychiatry Trainee working within the NHS who has experience of working with adults and children in a mental health setting. Her research interest is around supporting patients who are parents within NHS services. AI is an Asian male and is a Psychiatry Trainee working within the NHS who has experience of working with adults and children in a mental health setting. At the time of the study SdC and AI were working as Academic Clinical Fellows. ER identifies as a white female and Professor who has conducted extensive research on parental mental health and has a specific interest in communication with children about serious illness. LJD identifies as a white female and is a Consultant Clinical Psychologist who has worked extensively with parents and children. At the time of the study, she was working as a Clinical Academic. AD identifies as a white female with extensive experience of carrying out research on parental mental health; she works in an academic setting. Four authors have experience of delivering clinical services to adults and children within an NHS context (SdC, AI, LJD, AD); the team’s familiarity with the practical demands and pressures on frontline professionals working within the NHS may have helped establish a rapport with participants and potentially influenced interpretation of the results Participants were aware of the interviewers’ known interest in this area of work which may have led to participants feeling that their clinical practice was being scrutinized and thus inhibited any strong views about not communicating with children about parental illness. The team’s previous work around healthcare professionals’ role in family communication within a physical health setting (particularly around obstacles and facilitators) may have influenced their interpretation of the results within a mental health context.

## Results

### Participants

The participants were 19 MHPs who were currently working with adults under the care of adult mental health services in the NHS with a range of experience as outlined in **Tables 2**–**4**.

**Table 2:** Roles of adult mental healthcare professionals.

Participant ID	Professional Background	Number (n)
1, 2, 3, 8, 11, 12, 13, 14	Registered Mental Health Nurse	8
4, 5, 6	Consultant	3
7, 9, 15, 17	Clinical Psychologist	4
10	Occupational Therapist/Community Psychiatric Nurse	1
16, 18, 19	Junior Doctor	3

**Table 3:** Years of experience of adult mental healthcare professionals.

Years of experience	Number (n)
Over 30	4
Over 20	5
Over 10	6
Less than 10	4

**Table 4:** Current work setting of adult mental healthcare professionals.

Current work setting	Number (n)
Adult Inpatient	6
Adult Community	4
Adult Outpatient	2
Adult Liaison Psychiatry	2
Adult Community and Inpatient	2
Early Intervention Service (over 13 years old)	1
Adult Urgent Care Pathway	1
Ambulance Service	1

Participants reported identifying patients’ children to be a routine part of their role, but described a wide spectrum of experiences regarding talking to patients about family centred conversations. These are presented as five themes (**Table 5**).

**Table 5:** Themes and subthemes.

Theme	Sub theme
1 Identifying patients who are parents	
2 Family centred conversations in practice	
3 Absence of family centred conversations	i Conflict between family centred conversations and role as a MHP ii Beliefs that patients do not want MHPs’ involvement
4 Obstacles to family centred conversations	i Clinical setting ii Lack of specific skillset iii Emotional impact on MHPs and patients iv Uncertainty about the appropriateness of family centred conversation v Organisational culture
5 Facilitators to family centred conversations	

### Theme 1: identifying patients who are parents

All participants were unanimous in their perceived responsibility and “*duty of care*” to record information about their patients’ children in order to identify any safeguarding concerns. Participants reported routine recording of the children’s full names, dates of birth, and which schools they attended.

*“The admission pack. it’s got a check-list of questions that we will have to ask the patient and also the community team as well. So for instance, if they’ve got children, who their next of kin is and if they’re got pets, who is looking after the pets, who is looking after the children”* Participant 11 (Nurse)

Although identification of children was considered essential for safeguarding purposes, some participants reflected that they did not feel this was universal across their own practice and acknowledged a “*cultural template*” that influenced which patients they would check to see if they had children. These participants reflected they were less likely to ask younger or older patients, male patients, or those who were single and living alone.

*“Easy to forget it when it doesn’t seem like they would [have children]”* Participant 16 (Junior Doctor)

Most participants discussed their duties relating to safeguarding in terms of ascertaining the physical safety of any identified children, with only a minority reflecting on a broader consideration of children’s emotional wellbeing or longer-term safeguarding concerns such as the possible impact of poor mental health on parenting capacities.

*“The notes say that the children are okay, school says they are okay and they are with their grandparents at the moment, they are [not discussed or considered]”* Participant 4 (Consultant)

*“You’re naturally thinking of the risk to the child being neglected or abused, we were thinking this patient doesn’t come across as very unwell, she presents quite well, but when you dig a little, she seems to be spending most of the day staring vacantly into space, how can that be affecting her children if she’s sort of so vacant. I don’t think we took it to safeguarding.…. But it’s that subtle attachment stuff of presence and atunement, that was suffering rather than the sort of broad strokes ‘Daily Mail’ kind of level of stuff*” Participant 19 (Junior Doctor)

### Theme 2: family centred conversations in practice

A small number of participants expressed a clear view that supporting family centred conversations was part of their professional role.

“*I think it’s our role, because if it’s not our role, whose is it? You know, we are the experts, aren’t we? We are the ones who are advising them about their treatment, so part of it is also advising them on how best to support and help the people around them who also have effects on themselves from their mental illness”* Participant 18 (Junior Doctor)

Some of these participants had been trained in a “brief family intervention” through their role as mental health nurses on a specific inpatient ward. Participants described psychoeducation with family members about a patient’s mental illness as a significant aspect of this intervention, with sessions tailored to ensure they were age-appropriate for the children involved.

*“…we’d do some, you know some education around the condition, um, but personalise it so when mum’s not very well, so if it’s depression you might talk about the changes, the biological changes, and then how it makes mum feel so you might do something about thoughts, feelings, behaviours and the consequences of that. It would all depend on, you’d make it very much about that individual and that individual’s family”* Participant 2 (Nurse)

One participant described taking the initiative to develop a leaflet for a specific patient in response to the parent’s concern about how to talk to their children about her injuries. This participant reflected that self-harm wounds are more visible to children, whereas an overdose can be “disguised”.

*“…it was after I saw a woman who just didn’t know how to go home and explain to her children her wounds on her arm. So, me and one of the junior doctors did this leaflet. And really basic terminology … just explaining to kind of children really what self-harm is, and why you know, they’re not doing it because they are upset with the child…”* Participant 8 (Nurse)

A few participants reported that they had offered to support parents consider how to talk to their children, but these suggestions had not been taken up. Participants attributed this to a possible desire from parents to have some control over what or how information about their illness is shared with their children.

*“I’ve certainly offered…. I’ve said to them “if you’d like me to speak to your children like with you there, I could say a bit about how I’m supporting you or a bit about your health”…. but I’ve never been taken up on it, so I, I suspect that parents just feel more comfortable doing it on their own terms and maybe sharing as much or as little as they would like to….for some people, for 3 years I’ve going to their house every two weeks and say hi to their kids. the teenagers go back off into their room, and I’ve often wondered how much they know”* Participant 10 (Occupational Therapist/Nurse)

### Theme 3: absence of family centred conversations

Many participants reported that they had no experience of talking to patients about what their children had been told about the illness. Participants expressed a range of views about why this subject had not been initiated during consultations; some reported that they did not feel it was part of their role as a MHP (for example believing it to be best addressed by colleagues from other services) whereas others felt it was a subject which should be raised by parents themselves.

*“I think um there’s a general impression that that side belongs to social services. So, you can talk to people about how they feel, but as for facilitating contact or maintaining relationships no I don’t think ever. On inpatient wards in particular there’s the feeling it’s not our job, that belongs to somebody else”* Participant 9 (Clinical Psychologist)

“*if mum requests, okay we shall have that conversation, by all means, but other than that it’s not a role I think healthcare workers will play”* Participant 11 (Nurse)

*“Because often there’s a feeling …… that it’s not actually your job and you’re interfering a little bit, you know. Encroaching”* Participant 9 (Clinical Psychologist)

Some participants reflected that their patients had not asked them for help in talking to their children about their illness.

Interviewer*: “Has any parent ever asked you directly about how to speak to their children about their mental health?”*

Participant: *(Pause) “That’s a really interesting question and I think, think they never have.”* Participant 7 (Clinical Psychologist)

Other participants acknowledged that they had not previously considered talking to patients about what their children understood about their illness. However, during the study interview several reflected that this was an overlooked area in their current practice and expressed an openness to consider how such discussions might enhance clinical care.

*“I think that if I had been admitted to a ward and somebody didn’t fully acknowledge my role as a parent I would be devastated. It feels like a blind spot or a gap that we’re so used to that we just accept it, and that’s that. I don’t know where it comes from. I don’t know. But it [the gap] is there, and I think it’s not very good for care actually”* Participant 9 (Clinical Psychologist)

*“It is interesting to be asked [in research interview] about how I talk with parents about talking to their children about a diagnosis. And realising that’s not something I’ve necessarily given a lot of thought to”* Participant 17 (Clinical Psychologist)

#### Sub theme i: conflict between family centred conversations and role as a MHP

A few participants reported that they did not initiate conversations with patients about their children due to the sensitive nature of this area and their perception that it could adversely affect their therapeutic relationship.

“We’re getting into *some very difficult areas to talk to people about, and you also have to think about the therapeutic alliance and not rupturing it at an early stage by making people feel I’m inferring ‘What’s it like being a bad mother?’”* Participant 19 (Junior Doctor)

Others raised concerns that “*opening up*” the topic of children might lead to the identification of safeguarding concerns which would then need to be addressed and could also affect the relationship.

*“there’s always the question in my mind of.you know… ‘what exactly is gonna happen to it (regarding safeguarding concern)? I’m gonna have to pass this is on…. is anything really going to happen?’ Um, and you know potentially that can destroy a therapeutic relationship without any real impact, so say where something needs attention from social services….”* Participant 17 (Clinical Psychologist)

Participants who were medical doctors felt that a power dynamic existed between themselves and patients arising from their clinical responsibility for decisions about treatment or discharge from inpatient care. This was cited as inhibiting clinicians’ from engaging the patient in conversations about their parental experience and their children.

*“now and then, I have patients who appeal their section and I have to present, you know, at the tribunal and then at the tribunal you have to say things black and white, you know, and you have to say I think he is not well enough to go home because of the risks he poses to his children…”* Participant 4 (Consultant)

Others suggested that patients did not feel “the level of relationship [with their MHP] would never reach that trusting level” to raise the subject of communication with their children. This was attributed to the relative infrequency of outpatient appointments and recurrent changes in clinicians.

#### Sub theme ii: beliefs that patients do not want MHPs’ involvement with family centred conversations

Some participants suggested that patients may not ask MHPs with assistance in explaining their mental illness to their children as they “*know best*” about how to approach these conversations with their own children. In addition, participants reflected that patients may want to demonstrate that they are capable in their parenting role and that their approach may be better to that of MHPs.

*“It might be a sort of rugged individualism, so they are like ‘I’ll do it myself because they are my children’, it might (be) a sense that you wouldn’t ask a psychiatrist a question like that, they’re too head in the clouds and you get a silly answer about receptors”* Participant 19 (Junior Doctor)

*“I think patients think ‘I don’t need your help to do that’. Which is I suppose is like a protective, a protection, you know… ‘I don’t really need your help I can do this because I don’t want to be seen as weak’”* Participant 14 (Nurse)

Participants suggested that patients do not want to talk about their children as they may think that such conversations will precipitate other services becoming involved such as social services and a subsequent risk of their children being removed from their care.

*“I think one barrier that, you know, you’re seen as the other, as the professional, and there’s perhaps a reluctance to open up in case you get interference or unwelcome attention [from social services]”* Participant 9 (Clinical Psychologist)

*“Often people’s minds go straight to ‘Oh are you another professional that’s going to be proving that I’m not a good enough parent or gonna take my children away?’”* Participant 10 (Occupational Therapist/Nurse)

Another participant hypothesised that some patients who had experienced emotional deprivation during their early life might want and need to protect the therapeutic space for themselves, rather than making “*space”* for their children.

### Theme 4: obstacles to family centred conversations

Participants identified a range of obstacles which impeded them initiating discussions with patients about communication with children which are presented as 5 subthemes.

#### Sub theme i: clinical setting

At a structural level, all participants referenced the time constraints and pressure of clinical work within NHS settings. The specific location of professionals’ consultations with patients was often perceived as limiting what topics could be discussed. Participants working in the Emergency Department believed that this setting and the severity of the patient’s illness meant that it was inappropriate to initiate conversations with patients about talking to their children about mental illness.

*“… it’s not always the best place, A&E, to kind of focus too much on the family, especially when somebody I guess is in their own crisis and their family and children might not really have an impact on that so much”* Participant 8 (Nurse)

Another participant stated that the lack of available services for onward referral in the Emergency Department to follow up conversations about talking to children inhibited these conversations.

*“And I suppose who takes the baton for, um, not following up the OCD, but for how, how the child is coming to understand what’s going on for mum and how mum can understand boundaries and what she can and can’t, should and shouldn’t say to the 11 year old and, um, it’s who then takes on that baton”* Participant 5 (Consultant)

Participants who worked in mental health inpatient settings reported that the ward environment actively “*discourages young people visiting*”, through “*non-existent*” facilities for children and the “*dark, scary*” environment of wards. Participants reflected that the consequent invisibility of patients’ roles as parents meant communication with children was not addressed.

#### Subtheme ii: lack of specific skillset

Many participants reported a lack of skills, knowledge and confidence in initiating conversations with patients about their children. Participants felt they *lacked* specific training and had not had opportunities to observe colleagues modelling these skills.

*“I think I would find it very challenging actually … For me. I don’t think, I don’t think I know the words”* Participant 18 (Junior Doctor)

*“I think there’s a lot of anxiety and apprehension, I think it’s sometimes a challenge to get people to work with families full stop, let alone work with children. So, I think there’s a lack of training, um, and a lack of confidence and competence to, to work with um children and the parent they might be caring for”* Participant 2 (Nurse)

Several also commented that they were unsure what, if any, resources were available for themselves as clinicians, or to give to patients, about talking to their children which then prevented participants from signposting to reliable sources of information.

#### Subtheme iii: emotional impact on MHPs and patients

Many participants reflected the potential emotional impact on themselves as MHPs of thinking about patients’ children, or raising this topic area with their patients. These participants linked this to their reluctance to embark on conversations with patients about children.

*“…sometimes we want to escape these difficult conversations because it’s a difficult conversation to have…. maybe we’ve been, you know, kind of protecting ourselves from thinking about it, um and therefore we’ve just stopped thinking about it, about how we include children because it’s so close to heart”* Participant 18 (Junior Doctor)

Many participants highlighted the “*sensitive*” nature of initiating a conversation with patients about their children, which in part reflects the ongoing stigma associated with being a parent with mental illness. MHPs expressed concerns that this topic area might be counterproductive and potentially make patients “*feel worse*”. Consequently, these conversations were not something that they included as part of their routine practice.

*“it’s just hugely about like trying to break down stigma isn’t it which is I think what we all try to do all the time you know and you know trying to compare it to illnesses that you can see you know, you’d explain cancer wouldn’t you, you’d feel confident about explaining cancer”* Participant 8 (Nurse)

“*If it’s going to create more distress, you know, at the time they are with us it might not be appropriate*” Participant 11 (Nurse)

“*Separation is painful and bringing up children when it’s not possible for that parent to actually see them feels quite cruel*” Participant 9 (Clinical Psychologist)

Other professionals felt that talking about children could highlight to patients how their mental illness may impact directly on their parenting abilities and thus increase the risk of their child being affected.

“*Depressed people can feel sad about themselves.’not only do I have this awful condition but I could make myself feel worse about it by thinking about the effect it’s going to have on the generations’. That could be a bit of a downer*” Participant 19 (Junior Doctor)

For others, their decision not to mention children when talking to patients was on the presumption that patients may have “*a lot of sadness*” in their history, such as “*difficulties in their parenting role*” or may have “*missed the opportunit*y” to have children due to their mental illness.

#### Subtheme iv: uncertainty about the appropriateness of family centred conversations

Some participants reflected that they, or their colleagues, were unsure about the interaction between patient confidentiality, consent and communication with children. These participants felt that concerns about breaching patients’ rights to confidentiality inhibited taking the initiative to talk with children directly (during home or inpatient visits).

*“…am I allowed to talk to the child or not, would it be appropriate, do I need to take consent?”* Participant 6 (Consultant)

*“I think we still have in my opinion a misunderstanding of confidentiality. That sometimes it’s almost a knee jerk reaction you know when families contact the ward or relatives, um the main reaction is “oh no, we can’t share anything”* Participant 4 (Consultant)

Other participants expressed concern about the rationale for family centred conversations. Some suggested that patients might not want to talk with children, which they attributed to their desire to protect both themselves and their children from thinking about the diagnosis.

*“If you don’t say it maybe it’s not real”* Participant 18 (Junior Doctor)

Some participants were also unsure about how helpful it would be for children to know about their parent’s mental illness, including worries that this might result in children feeling inappropriately responsible for their parent.

*“I wonder if that’s a concern that would be held more broadly like ‘is it appropriate for parents to talk about a mental health issue to their children?’. What would be the benefits or costs of that?”* Participant 17 (Clinical Psychologist)

*“…it’s always been a concern that you know, why does the child have to take the responsibility and the impact that the child might be having taking that responsibility? …. is it okay? Can the child bear that sort of you know responsibility without having any emotional impact on them?… there’s always a, always a slight degree of, I wouldn’t say hesitancy, but um you know concern you know ‘is this the right thing to do?’”* Participant 6 (Consultant)

#### Sub theme v: organisational culture

A few participants reflected on their perception of the culture within their place of work in adult mental health services, which they felt was not conducive for considering family centred conversations.

*“There’s like a switch when you treat adults, you just go into almost the individual … is the whole thing you treat and you don’t, you’re not, not the teams, nor the services are built to think ‘well hang on, there’s these children, what do we do about this?’”* Participant 18 (Junior Doctor)

Another participant commented on the challenge of trying to shift organisational priorities and agendas to maintain attention on family centred initiatives.

*“…I think a focus changes on a lot of stuff, you know we had big drive around ‘Think Family’ a few years ago and we had a ‘Think Family’ champion, um, and then I guess the drive changes around, I don’t know, ‘physical wellbeing alongside mental health’, things drop off don’t they, you can’t retain it all”* Participant 8 (Nurse)

*“I think there’s a degree of helplessness around it as well you know, you just um, it seems so small, but you can’t do anything about it. Um and it’s so important for that child and that family. And you, it’s almost like you’re working against a huge system”* Participant 9 (Clinical Psychologist)

### Theme 5: facilitators to family centred conversations

Participants identified a number of areas which could help embed family centred conversations into practice. Many participants highlighted their need for specific training around supporting these conversations, noting its absence in their professional education; one participant suggested this should be mandatory. Participants thought it would be useful to include both the latest evidence about children’s needs when a parent has a mental illness to help with confidence, and practical guidance including suggested words and phrases. Several MHPs indicated that workbooks or leaflets would be useful to introduce the subject with patients.

*“There isn’t any on a specific training around speaking to families. It’s confidence around asking difficult questions … Um. Training, could training help with that? I don’t know. I think it comes with experience doesn’t it. A bit. Um. And not being fearful to ask”* Participant 8 (Nurse)

*“We’re not trained in any special way. Maybe it would be helpful to have some very specific training on that area so that you know feel armed with the right tool to go in there and say the right things….or having something we take to the parent and say ‘have a look at this, this is a helpful guide on how to communicate with your little ones about how you feel and what’s happening to you’”* Participant 13 (Nurse)

Furthermore, many participants reported a lack of available resources, such as age-appropriate content for parents and children, or specific guidelines for clinicians, as a barrier to engaging in these conversations.

*“…they (patients) have asked for ‘how do I explain it?’, and um and I think that’s the one of the biggest problems because there isn’t a great wealth of resources…”* Participant 2 (Nurse)

In contrast, other participants described either current or historical initiatives which had contributed to their knowledge around family focused practice and awareness of resources to facilitate children’s understanding of mental illness. Several participants referenced the “Think Family” government-commissioned approach to “raise awareness of teams and clinicians to be thinking about the children in households where there were parents”. Participants reported that this project had prompted them to think about providing more “*child friendly environments*” and spaces for patients to interact with their children, in addition to accessing leaflets and novels aimed at different age groups to facilitate children’s understanding of parental mental illness. However, they reflected that this initiative had not resulted in sustained change to practice.

*“As part of the ‘Think Family’ job, we provided all the wards with some resources for you know literature that they could help young children understand from the age of sort of 5, from story books through to sort of teenage children where you know sort of main stream novels that were very much helping them understand why their parent may be having in a way that they are or experiencing what they are experiencing”* Participant 1 (Nurse)

A small number of participants reported that they perceived colleagues to have an incomplete understanding of the impact of parental mental illness on children, which, if addressed, could contribute to positive change in how discussions about children are viewed within adult mental health services. For example, it was suggested that this knowledge might encourage a broader consideration of what constitutes a safeguarding assessment. Others indicated that a requirement to record family centred conversations as part of routine processes might help to embed this aspect of care.

*“I think what is potentially lacking is … they’re not seeing the potential harm … to those children. Erm. Because it’s not a very sort of high profile, safeguarding concern.”* Participant 1 (Nurse)

*“What is that data out there, the research out there to show the impact of parental mental health? And that kind of gives them better understanding and accordingly prioritise that. Because I think perhaps it is not the highest of the priority at this stage. But that only happens if there is you know um information, knowledge and it is felt that that is the need”* Participant 6 (Consultant)

A few participants who had worked on the same inpatient ward described providing a brief family intervention as part of routine care. This initiative had been established by a senior nurse who had drawn on their previous experience of working with patients’ children in the community to prioritise and establish this aspect of care within the hospital.

## Discussion

Most MHPs in this study believed they had an important role in identifying children in their adult patient population, but this was primarily for safeguarding. Furthermore, any consideration of children was generally limited to their physical safety and not the emotional impact parental mental illness could have on children. Given this limited engagement with patients’ parental role, it is unsurprising that many MHPs described an absence of conversations about how to support parents to communicate with their children, and a lack of clarity about whether this would be appropriate or helpful for patients and their families.

Identification of patients’ children is important as these young people are at the greatest risk of developing mental illness themselves (5, 7). The results of this study are regrettably consistent with previous research indicating the invisibility of children of adult mental health patients (30, 47, 48) and that progress is still needed to achieve universal screening of patients. Recognition of patients’ role as a parent enables patients to access support around being a parent, which can improve patients’ outcomes and have benefits for their wider family (49). Furthermore, the identification of children affected by parental mental illness enables consideration of their own emotional needs and onward referral to mental health services or other appropriate interventions as required (50–54). Participants’ universal commitment to the ‘safeguarding’ of patients’ children highlights an opportunity to enhance practice through professional education about the broader aspects of safeguarding, rather than the current limited focus on children’s physical safety alone.

Participants reported they often based their decision to ask about children on their patients’ age, gender, and relationship status, which fails to reflect the complexity of family relationships or caregiving roles (55). Some participants were also influenced by the severity of the mental illness and the clinical setting. The importance of identifying children as part of standard practice is reflected in service-evaluation audit tools developed in Northern Ireland and Australia (56, 57). By facilitating routine identification of patients as parents, services would foster an environment in which barriers to other elements of family focused practiced could be dismantled. This could include ensuring there is a suitable environment or family space for children to visit their parent during an admission. A suitable, ringfenced space for families and children is enshrined in international mental health guidelines (58) and must be addressed and prioritised by services (59). This could also offer opportunities for MHPs to engage with adults in the wider family system (e.g. the ‘well’ parent or grandparent) around communicating with children about their parent’s illness.

Embedding family centred conversations into routine practice requires an understanding of MHPs’ perceptions of their role in this process. Participants in our study echoed the well-documented concern of patients that they might be judged as unable to care for their children (43); furthermore many expressed a belief that patients would not welcome discussions about their children and that it had the potential to cause patients’ distress, or negatively impact on their therapeutic relationship (60, 61). Other work has shown that professionals prioritise their patients’ problems, believing that other services are more appropriate to address the needs of any children; ‘*patient first, parent second*’ (62). Participants in this study did not demonstrate an awareness of the desire of family members and carers to have their relationships and responsibilities to children to be acknowledged and supported (43, 63). Parents want ongoing support for themselves and their children (30), including how to talk to children about their diagnosis (64, 65). This is also reflected in children’s wishes for information about their parent’s condition to help them understand illness-related behaviour (48, 66) with a preference to receive this from professionals (67). A ‘co-operative’ and collaborative partnership between patients and professionals is central to facilitating these important conversations about patients’ children and thereby improving patient and family outcomes (43).

The study identified several facilitators for professionals 1) widespread dissemination of the research and evidence of the effect of family centred conversations on child and family outcomes 2) specific training of how to initiate these discussions with patients and how to answer common questions and concerns 3) resources for patients to help guide conversations with their children 4) support from managers and institutions. The universal request for training and specific practical resources surrounding family centred conversations by participants was thought to increase individual knowledge and confidence. Increasing MHPs’ awareness of the benefits of identifying patients who are parents for both patient and child may help allay MHPs’ concerns about creating distress or disruption to their therapeutic relationships. Our findings regarding the emotional impact of sensitive conversations with patients about their children on healthcare professionals has been previously reported in both the physical and mental health literature (42, 68). This highlights the importance of considering how MHPs are supported not only practically, but crucially *emotionally*, when initiating family centred conversations.

Whilst increasing MHPs’ awareness and knowledge of patient and children’s needs through training and resources may help promote family centred conversations in routine practice (69), this is unlikely to be adequate without systemic change (47, 70). Initiatives such as “Think Family” were recognised by participants to be an effective method to motivate staff to discuss children with their patients and thus could be helpful to promote awareness. However, exposure to this model of practice was not universal across participants and appeared to be held only by a particular team within the NHS trust. This reiterates the essential requirement for training and innovation to be supported by organisation change and leadership to ensure sustainability (42, 43). Participants in this study highlighted the importance of a coordinated approach across different parts of the healthcare system (for example the Emergency Department, Inpatient and Outpatient services) so that once a conversation about children had been initiated with patients, these MHPs can feel confident that other parts of the system will respond to any identified concerns. Families experiencing parental mental health difficulties can have multiple touchpoints with different professionals such as GPs, social workers and the police, who all could play a role in facilitating what children know and understand about parental illness. These contacts may provide further opportunities to highlight the importance of communicating with children about their parent’s psychological well-being. Shared resources for professionals working across these different services (including practical suggestions of words and phrases to initiate family centred conversations and use with children) would ensure consistency of messaging and encourage a joined-up, cross generational approach.

## Strengths and limitations

This study is the first paper to explore the attitudes and beliefs of MHPs working in both inpatient and outpatient settings in the UK which focuses *specifically* on the topic of communicating with children about parental mental illness. It builds on existing research with healthcare professionals working with patients who have a serious physical illness to engage in conversations about their conditions with their children (68, 71). The participants included a broad range of MHPs from different professional backgrounds and experience currently working within mental health services.

The limitations of this study include being based in a single NHS mental health trust in the UK and thus results may not be representative across all NHS settings. Data was not collected on the parental role of participants and thus the impact of this experience on the results could not be explored. MHPs’ reported practice was not triangulated with clinical records or their patients’ experience of care relating to their potential role as a parent. The use of convenience sampling may have introduced a bias in the study participants towards those have an existing interest in parents with mental health difficulties. However, the accounts of MHPs regarding the identification of parents aligns with other research on this area.

## Conclusions

The results of this study highlight a disconnect between families’ need for information and support about parental mental illness and MHPs’ perceptions and clinical practice in routine care. Facilitating family centred conversations could contribute to addressing the needs of children whose parents have a mental illness. This may help reduce their vulnerability to future mental illness and the spiralling global mental health crisis. To achieve this goal, professionals need specific training and resources which is supported at the organisational and policy level.

## Data Availability

The raw data supporting the conclusions of this article will be made available by the authors, without undue reservation.
